# Supplementary material for: An evolutionary mechanism to assimilate new nutrient sensors into the mTORC1 pathway
Source: Nat Commun. 2024 Mar 21;15:2517. doi: 10.1038/s41467-024-46680-3 (PMC10957897; doi:10.1038/s41467-024-46680-3)
Supplement: Supplementary file 1 — Supplementary Information [file 41467_2024_46680_MOESM1_ESM.pdf]

An evolutionary mechanism to assimilate new nutrient sensors into the mTORC1 pathway

Authors: Grace Y. Liu[1,2,3]*, Patrick Jouandin[4,5,6], Raymond E. Bahng[1,2,3], Norbert Perrimon[4,5]*, David M. Sabatini[7*]

Affiliations:

[1] Whitehead Institute for Biomedical Research and Massachusetts Institute of Technology, Department of Biology; 455 Main Street, Cambridge, Massachusetts 02142, USA.

[2] Department of Biology, Massachusetts Institute of Technology; 77 Massachusetts Avenue, Cambridge, Massachusetts 02139, USA. dmsabatini.lab@gmail.com

[3] Koch Institute for Integrative Cancer Research and Massachusetts Institute of Technology, Department of Biology; 77 Massachusetts Avenue, Cambridge, Massachusetts 02139, USA.

[4] Department of Genetics, Blavatnik Institute, Harvard Medical School; Boston, MA 02115, USA.

[5] Howard Hughes Medical Institute, Harvard Medical School; Boston, MA 02115, USA.

[6] Present address: Institut de Recherche en Cancérologie de Montpellier, Inserm U1194-UM-ICM; Campus Val d'Aurelle, F-34298 Montpellier Cedex 5, France

[7] Institute of Organic Chemistry and and Biochemistry, Flemingovo n. 2, 166 10 Praha 6, Czech Republic

*Corresponding author. Email: grace.yun.liu@berkeley.edu, perrimon@genetics.med.harvard.edu, david.sabatini@uochb.cas.cz

a

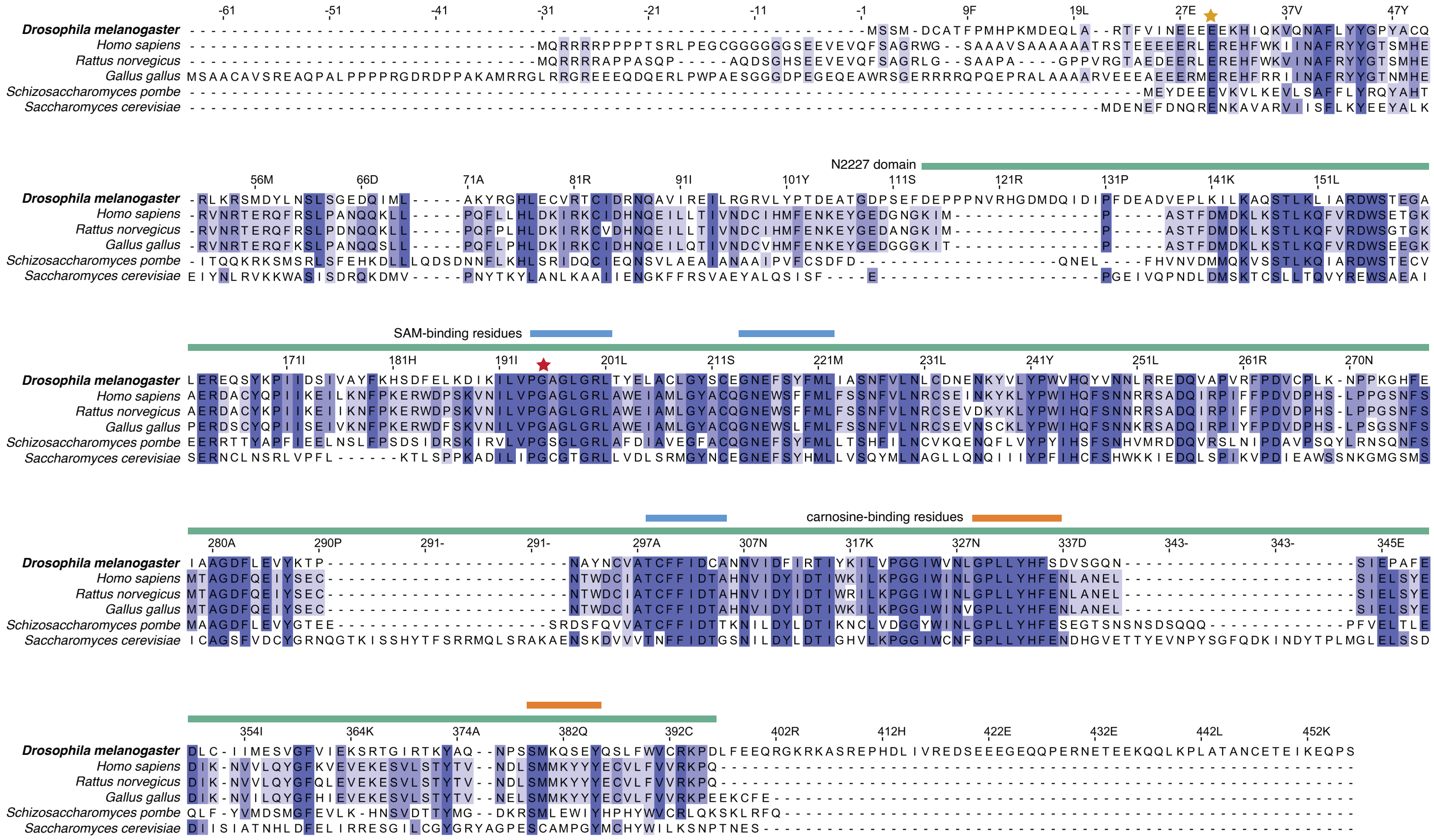
Drosophila melanogaster
Homo sapiens
Rattus norvegicus
Gallus gallus
Schizosaccharomyces pombe
Saccharomyces cerevisiae
N2227 domain
SAM-binding residues
carnosine-binding residues
-61 -51 -41 -31 -21 -11 -1 9F 19L 27E 37V 47Y
56M 66D 71A 81R 91I 101Y 111S 121R 131P 141K 151L
171I 181H 191I 201L 211S 221M 231L 241Y 251L 261R 270N
280A 290P 291- 291- 297A 307N 317K 327N 337D 343- 343- 345E
354I 364K 374A 382Q 392C 402R 412H 422E 432E 442L 452K

Supplementary Figure 1: Multiple sequence alignment of Unmet homologs.

(**a**) Sequence alignment of Unmet and homologs from various organisms. Residues are colored by percent identity. Numbering refers to the positions in the *Drosophila melanogaster* Unmet protein sequence (GenBank accession number AAG22387.1). N2227 domain boundaries (green) were annotated based on the PFAM database; approximate metabolite-binding regions for SAM (blue) and carnosine (orange) were identified by finding residues within 3Å of each metabolite in a crystal structure of human CARNMT1 bound to sinefungin and carnosine (PDB: 5YF1). Residues important for the dGATOR2- and SAM-binding capacities of Unmet are marked with yellow and red stars, respectively, at E30 and G195.

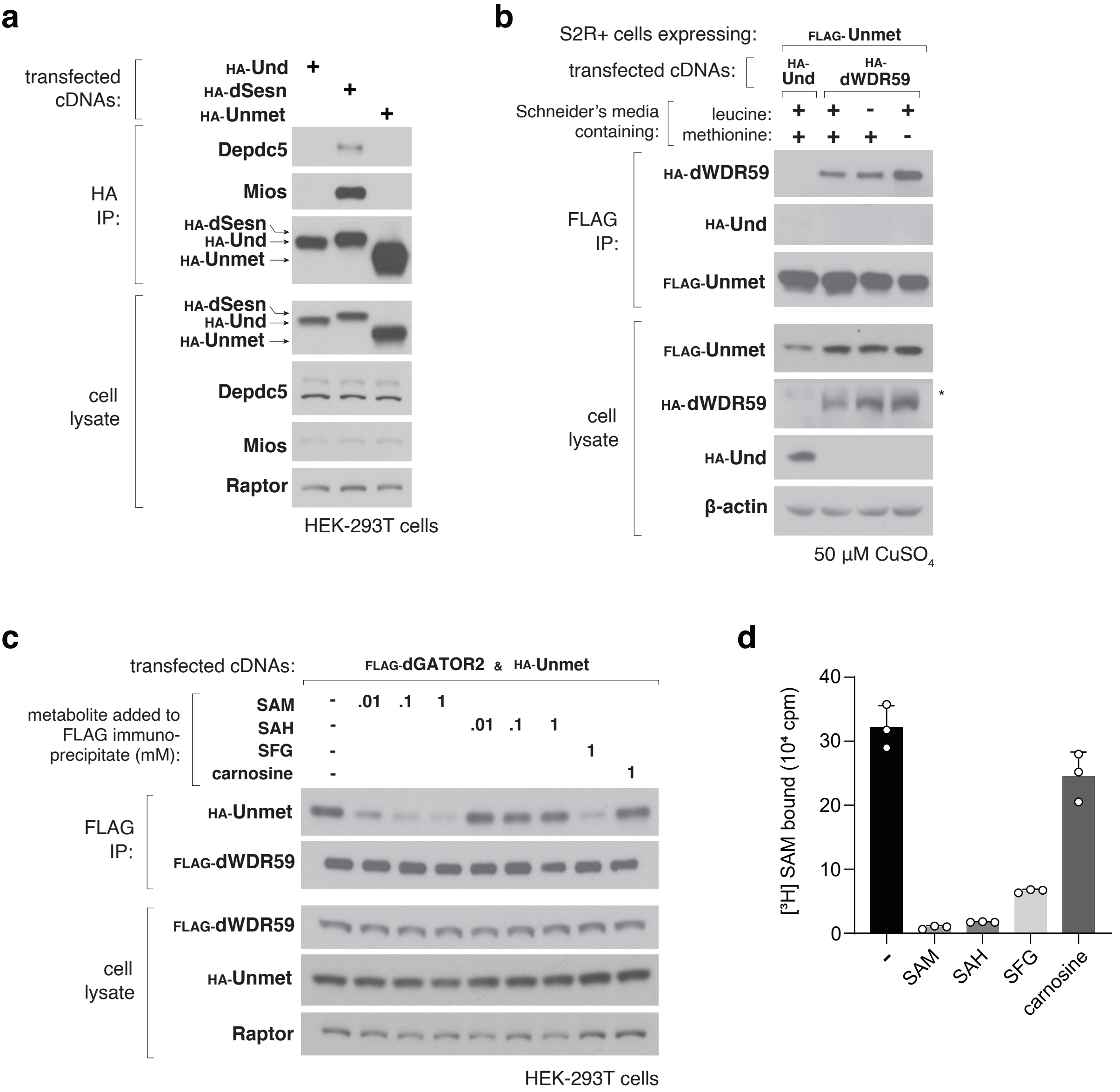

a
transfected cDNAs:
HA-Und
HA-dSesn
HA-Unmet
HA IP:
Depdc5
Mios
HA-dSesn
HA-Und
HA-Unmet
cell lysate
HA-dSesn
HA-Und
HA-Unmet
Depdc5
Mios
Raptor
HEK-293T cells
b
S2R+ cells expressing:
FLAG- Unmet
transfected cDNAs:
HA-Und
HA-dWDR59
Schneider's media containing:
leucine:
methionine:
FLAG IP:
HA-dWDR59
HA-Und
FLAG-Unmet
cell lysate
FLAG-Unmet
HA-dWDR59
HA-Und
β-actin
50 µM CuSO4
c
transfected cDNAs:
FLAG-dGATOR2 & HA-Unmet
metabolite added to FLAG immuno-precipitate (mM):
SAM - .01 .1 1
SAH - .01 .1 1
SFG - 1
carnosine - 1
FLAG IP:
HA-Unmet
FLAG-dWDR59
cell lysate
FLAG-dWDR59
HA-Unmet
Raptor
HEK-293T cells
d
[3H] SAM bound (10⁴ cpm)
40
30
20
10
0
-
SAM
SAH
SFG
carnosine

Supplementary Figure 2: The fly-specific interaction between Unmet and dGATOR2 is regulated by SAM but not SAH or carnosine.

(**a**) Recombinant Unmet does not interact with the endogenous human GATOR1 or GATOR2 complexes. Anti-HA immunoprecipitates were collected from HEK-293T cells transfected with the indicated cDNAs in expression vectors and were analyzed alongside cell lysates by immunoblotting for the levels of the indicated proteins and epitope tags. HA-Und served as a negative control. Depdc5 and Mios were used as a representative components of GATOR1 and GATOR2, respectively.
(**b**) Methionine starvation enhances the interaction between Unmet and dGATOR2 in fly cells. S2R+ cells expressing copper-inducible FLAG-tagged Unmet from the endogenous locus were transfected with HA-Und or HA-dWDR59 in constitutive expression vectors and induced with 50 μM $CuSO_4$ for 72 hours. Cells were then cultured in full, leucine-free, or methionine-free Schneider's media for 2 hours. FLAG immunoprecipitates and cell lysates were analyzed as in Fig. 2d.
(**c**) The Unmet-dGATOR2 complex is disrupted by 100 μM of SAM or 1 mM of SFG but not by 1 mM of SAH or carnosine. The experiment was performed and analyzed as in Fig. 2e.
(**d**) Unmet binds to SAM, SAH, and SFG. Binding assays were performed with 10 μg purified FLAG-Unmet incubated with 5 μM [^{3}H]SAM and 1 mM of unlabeled SAM, SAH, SFG, or carnosine. Values for each point represent the means ± s.d. from three independent replicates.

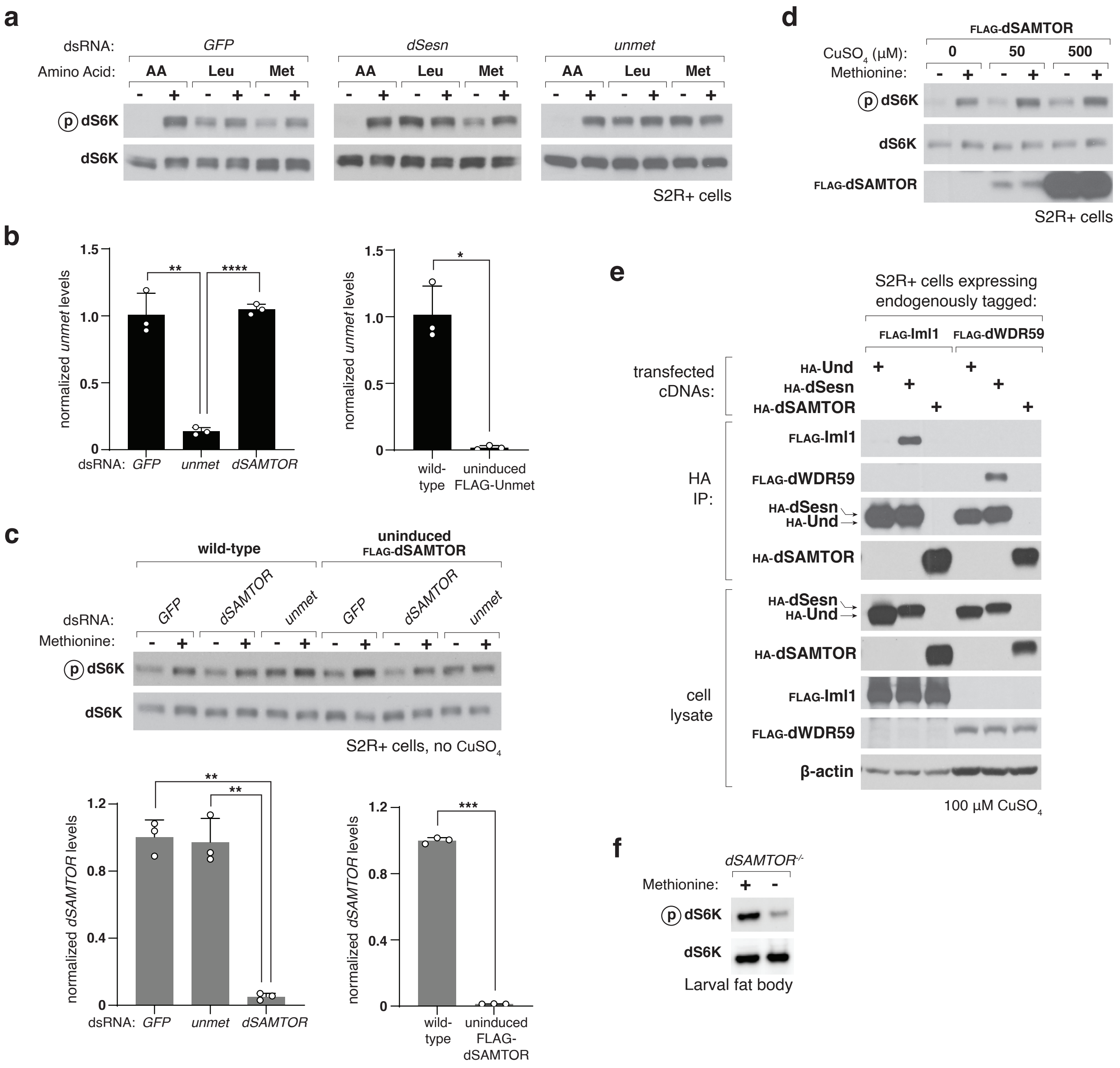
a
dsRNA: GFP dSesn unmet
Amino Acid: AA Leu Met
dS6K
S2R+ cells
b
normalized unmet levels
dsRNA: GFP unmet dSAMTOR
wild-type
uninduced FLAG-Unmet
c
wild-type
uninduced FLAG-dSAMTOR
dsRNA: GFP dSAMTOR unmet
Methionine:
S2R+ cells, no CuSO4
normalized dSAMTOR levels
uninduced FLAG-dSAMTOR
d
FLAG-dSAMTOR
CuSO4 (μM): 0 50 500
Methionine:
S2R+ cells
e
S2R+ cells expressing endogenously tagged:
FLAG-Iml1 FLAG-dWDR59
transfected cDNAs:
HA-Und
HA-dSesn
HA-dSAMTOR
HA IP:
cell lysate
β-actin
100 μM CuSO4
f
dSAMTOR-/-
Larval fat body

Supplementary Figure 3: Unmet, not dSAMTOR, signals methionine sufficiency to the dTORC1 pathway.

(**a**) The dTORC1 pathway is resistant to methionine starvation in Unmet-depleted cells. S2R+ cells were transfected with dsRNAs targeting a control mRNA (*GFP*), *dSesn* mRNA, or *unmet* mRNA. dsRNA-treated cells were then starved of the indicated amino acids for 90 minutes or starved and restimulated for 15 min. Cell lysates were analyzed by immunoblotting for the phosphorylation states and the levels of dS6K.
(**b**) mRNA levels of *unmet* in S2R+ cells treated with the indicated dsRNAs. In the absence of copper induction, *unmet* mRNA levels in S2R+ cells expressing FLAG-Unmet from a metallothionein promoter at the endogenous locus are comparable to those of cells with a knockdown of *unmet*. Reported values reflect the mean ± s.d. of three biological replicates of ΔΔCt values, using α-tubulin as an internal standard. Two-sided Student's t-test; from left to right: $^{**}P = 9.7 \times 10^{-3}$; $^{****}P < 1.4 \times 10^{-5}$; $^{*}P = 1.5 \times 10^{-2}$.
(**c**) Depleting *unmet* abolishes dTORC1 sensitivity to methionine starvation, while depleting *dSAMTOR* has no effect. Wild-type S2R+ cells or S2R+ cells expressing copper-inducible FLAG-tagged dSAMTOR from the endogenous locus were transfected with the indicated dsRNAs in the absence of copper. dsRNA-treated cells were starved of methionine as in (a), and cell lysates were analyzed by immunoblotting for the phosphorylation states and levels of dS6K. cDNA from transfected cells was synthesized and analyzed by qPCR. Two-sided Student's t-test; from left to right: $^{**}P = 2.8 \times 10^{-3}$; $^{**}P = 7.0 \times 10^{-3}$; $^{***}P = 1.1 \times 10^{-4}$. Error bars represent the s.d. around the mean of three independent samples.
(**d**) dSAMTOR expression does not impact dTORC1 activity. S2R+ cells expressing a copper-inducible FLAG-tagged dSAMTOR from the endogenous locus were incubated with the indicated concentrations of $CuSO_4$ for 72 hours. Cells were then starved of methionine, and lysates were analyzed as in (a).
(**e**) dSAMTOR does not interact with the dGATOR1 or dGATOR2 complexes. Anti-HA immunoprecipitates were prepared from S2R+ cells bearing endogenous FLAG knock-in tags at either the Iml1 (dGATOR1) or the dWDR59 (dGATOR2) locus, transfected with the indicated cDNAs in copper-inducible metallothionein expression vectors. Following 48-hour induction with 100 µM $CuSO_4$, cell lysates and immunoprecipitates were analyzed by immunoblotting for levels of the relevant epitope tags.
(**f**) In the larval fat bodies of *dSAMTOR*$^{-/-}$ flies, the dTORC1 pathway remains sensitive to methionine starvation. *dSAMTOR*$^{-/-}$ L3 larvae were transferred to either full or methionine-free holidic diets for 24 hours. Dissected fat bodies were crushed and analyzed by immunoblotting for the phosphorylation states and the levels of dS6K.

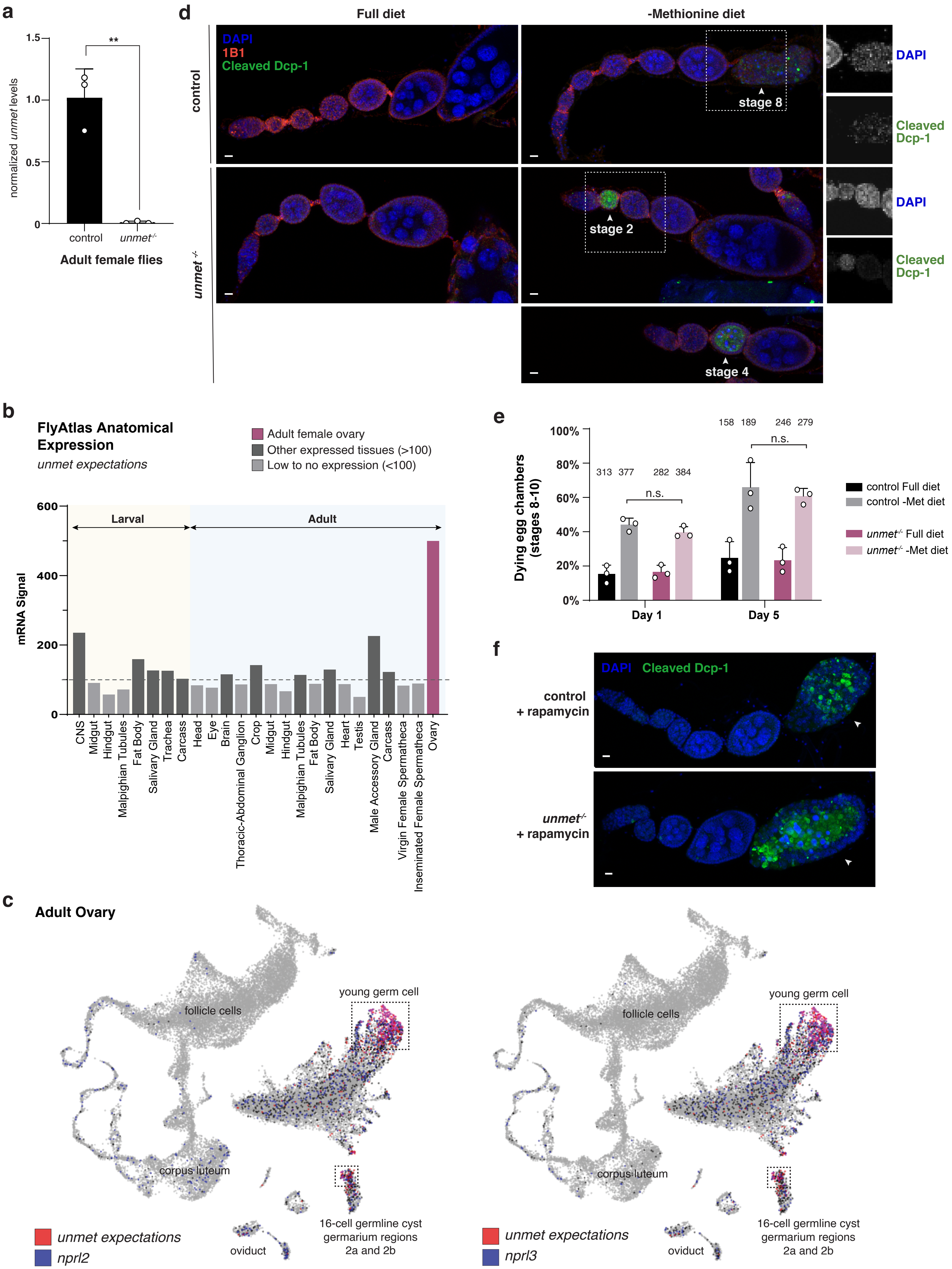

a
1.5
1.0
0.5
0
**
normalized unmet levels
control
unmet-/-
Adult female flies
d
Full diet
-Methionine diet
control
unmet -/-
DAPI
1B1
Cleaved Dcp-1
stage 8
stage 2
stage 4
DAPI
Cleaved Dcp-1
b
FlyAtlas Anatomical Expression
unmet expectations
Adult female ovary
Other expressed tissues (>100)
Low to no expression (<100)
600
400
200
0
mRNA Signal
Larval
Adult
CNS
Midgut
Hindgut
Malpighian Tubules
Fat Body
Salivary Gland
Trachea
Carcass
Head
Eye
Brain
Thoracic-Abdominal Ganglion
Crop
Midgut
Hindgut
Malpighian Tubules
Fat Body
Salivary Gland
Heart
Testis
Male Accessory Gland
Carcass
Virgin Female Spermatheca
Inseminated Female Spermatheca
Ovary
e
100%
80%
60%
40%
20%
0%
Dying egg chambers (stages 8-10)
313 377 282 384
158 189 246 279
n.s.
n.s.
Day 1
Day 5
control Full diet
control -Met diet
unmet-/- Full diet
unmet-/- -Met diet
f
DAPI Cleaved Dcp-1
control + rapamycin
unmet-/- + rapamycin
c
Adult Ovary
follicle cells
young germ cell
corpus luteum
oviduct
16-cell germline cyst germarium regions 2a and 2b
unmet expectations
nprl2
nprl3

Supplementary Figure 4: *unmet* is expressed in young germ cells and acts on early rather than vitellogenic egg chambers.

(**a**) $unmet^{-/-}$ flies with CRISPR-Cas9-mediated deletion of the gene locus do not express any detectable *unmet* mRNA. Reported values reflect the mean ± s.d. of three biological replicates of ΔΔCt values, using α-tubulin as an internal standard. Two-sided Student's t-test; $^{**}P = 8.5 \times 10^{-3}$.

(**b**) Anatomical expression of *unmet* based on the Fly Atlas.

(**c**) Single-cell expression map for GATOR1 components *nprl2* and *nprl3* (blue) in the adult ovary, plotted against the expression map for *unmet* (red). HVG UMAP display of single-cell RNA-seq expression data from the Fly Cell Atlas. *nprl2* and *nprl3* expression overlaps with the expression of *unmet* in young germ cells and the germline cyst (purple).

(**d**) Ovarioles from female flies cultivated on the indicated diets for five days and labeled with DAPI (blue) and antibodies for hu-li tai shao actin-associated 1B1 (red), and cleaved *Drosophila* caspase 1 (cleaved Dcp-1 Asp216, green). Note that the degenerating egg chambers (white arrows) contain condensed DNA staining for pyknotic nuclei and are positive for cleaved Dcp-1. Scale bar, 10 µm. $unmet^{-/-}$ flies fed a methionine-free diets display degenerating early egg chambers (stages 2-7).

(**e**) Mid-stage (8-10) vitellogenic egg chambers from flies starved of methionine undergo apoptosis at identical rates between $unmet^{-/-}$ and control flies. Percentage of stage 8-10 egg chambers undergoing cell death were recorded for each genotype and dietary condition. Two-way ANOVA followed by Tukey's multiple comparison test; from left to right: adjusted $P = 0.88$; $P = 0.83$; n.s., not significant. Error bars represent the s.d. around the mean of three independent experiments. Bars are labeled with number of stage 8-10 egg chambers analyzed for each condition.

(**f**) In both $unmet^{-/-}$ and control flies, rapamycin treatment induces degeneration of mid-stage (8-10) egg chambers, while early egg chambers (1-7) remain intact. Ovaries from flies cultured for five days on a full diet containing 10 µM rapamycin were labeled with DAPI (blue) and cleaved Dcp-1 Asp216 (green). Note that the degenerating stage 8-10 egg chambers (white arrows) are positive for cleaved Dcp-1. Scale bar, 10 µm.

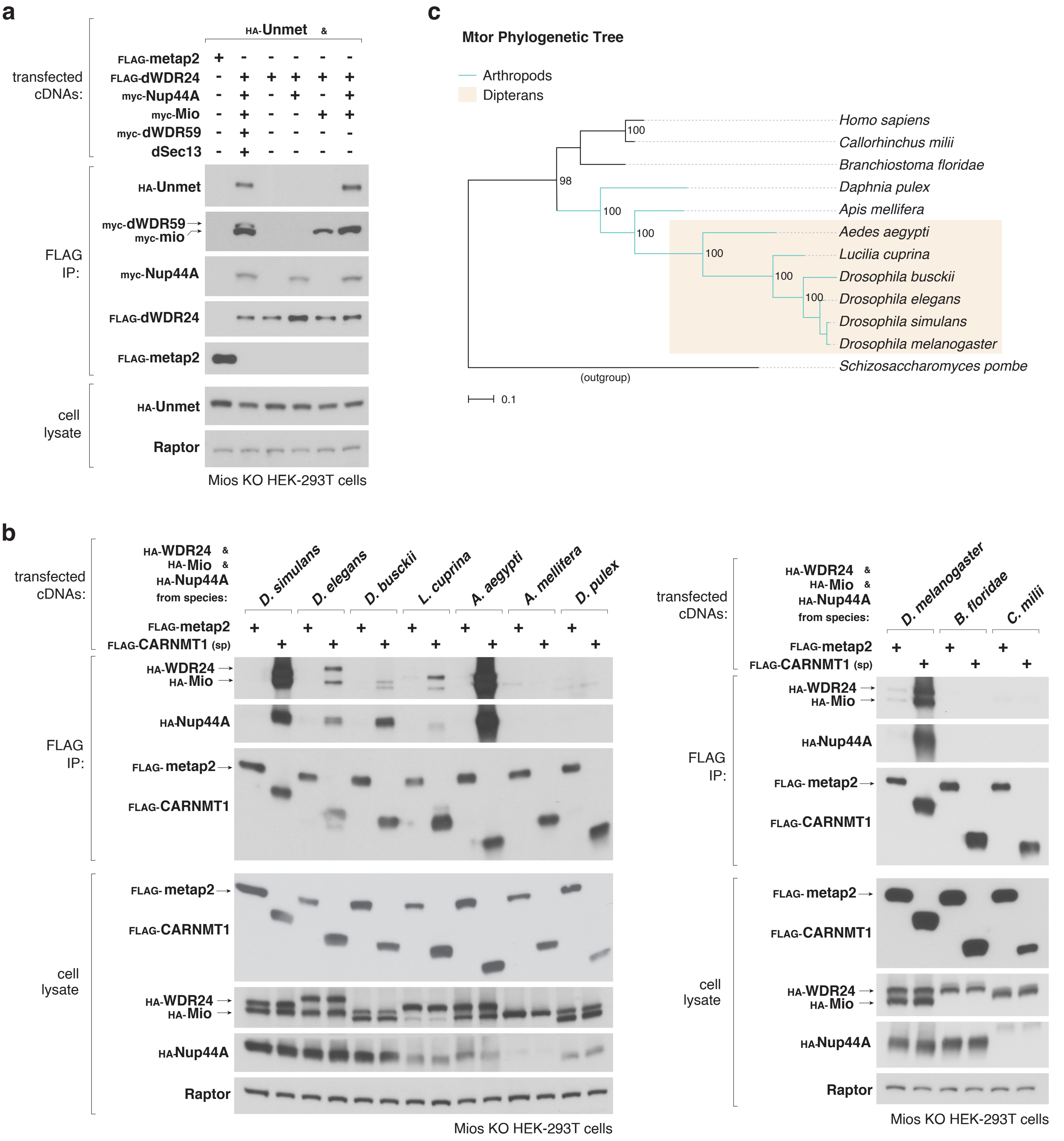
a
transfected cDNAs:
HA-Unmet &
FLAG-metap2
FLAG-dWDR24
myc-Nup44A
myc-Mio
myc-dWDR59
dSec13
FLAG IP:
HA-Unmet
myc-dWDR59
myc-mio
myc-Nup44A
FLAG-dWDR24
FLAG-metap2
cell lysate
HA-Unmet
Raptor
Mios KO HEK-293T cells
c
Mtor Phylogenetic Tree
Arthropods
Dipterans
Homo sapiens
Callorhinchus milii
Branchiostoma floridae
Daphnia pulex
Apis mellifera
Aedes aegypti
Lucilia cuprina
Drosophila busckii
Drosophila elegans
Drosophila simulans
Drosophila melanogaster
Schizosaccharomyces pombe
(outgroup)
0.1
b
transfected cDNAs:
HA-WDR24 &
HA-Mio &
HA-Nup44A
from species:
D. simulans
D. elegans
D. busckii
L. cuprina
A. aegypti
A. mellifera
D. pulex
FLAG-metap2
FLAG-CARNMT1 (sp)
FLAG IP:
HA-WDR24
HA-Mio
HA-Nup44A
FLAG- metap2
FLAG-CARNMT1
cell lysate
FLAG- metap2
FLAG-CARNMT1
HA-WDR24
HA-Mio
HA-Nup44A
Raptor
Mios KO HEK-293T cells
D. melanogaster
B. floridae
C. milii
Mios KO HEK-293T cells

Supplementary Figure 5: Dipteran GATOR2 acquired a novel interaction with Unmet.

(**a**) The dGATOR2 components dWDR24, Mio, and Nup44A form a minimal complex that is sufficient to co-immunoprecipitate Unmet. HEK-293T cells lacking the human GATOR2 complex protein Mios were co-transfected with the indicated cDNAs, and anti-FLAG immunoprecipitates and cell lysates were analyzed by immunoblotting for the indicated proteins and epitope tags.
(**b**) Dipteran GATOR2 complexes are capable of interacting with cognate CARNMT1 proteins from the same species. HEK-293T cells lacking human Mios were co-transfected with cDNAs encoding homologs of WDR24, Mio, and Nup44A from the indicated species, as well as either the negative control protein metap2 or the homolog of CARNMT1 from the indicated species. Anti-FLAG immunoprecipitates were analyzed as in (a).
(**c**) Species tree constructed using mTOR protein sequences from 12 species. Node labels indicate bootstrap support values. Scale bar, 0.1 substitutions per site. A phylogenetic tree constructed from WDR24 protein sequences displays an identical topology.

a

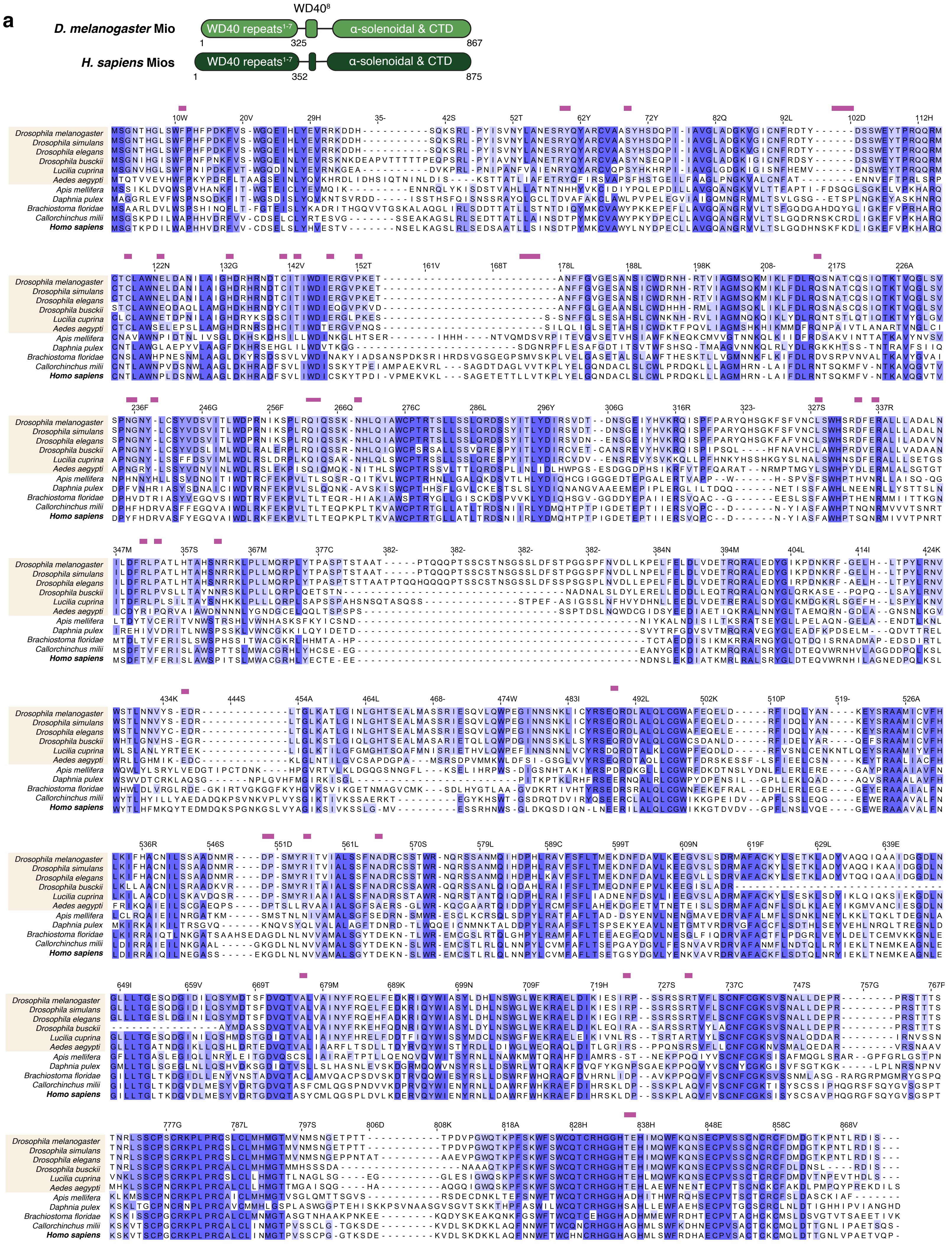

b

code	Mio chimera boundaries	chimera description
Mio D	1-867 fly	
Mio H	1-875 human	
Mio DnHc	1-325 fly, 353--875 human	fly WD40 repeats, human C-terminus
Mio HnDc	1-352 human, 326-867 fly	human WD40 repeats, fly C-terminus

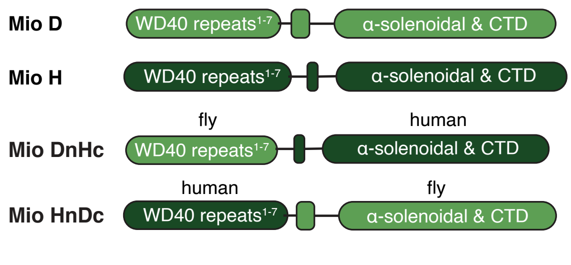

c

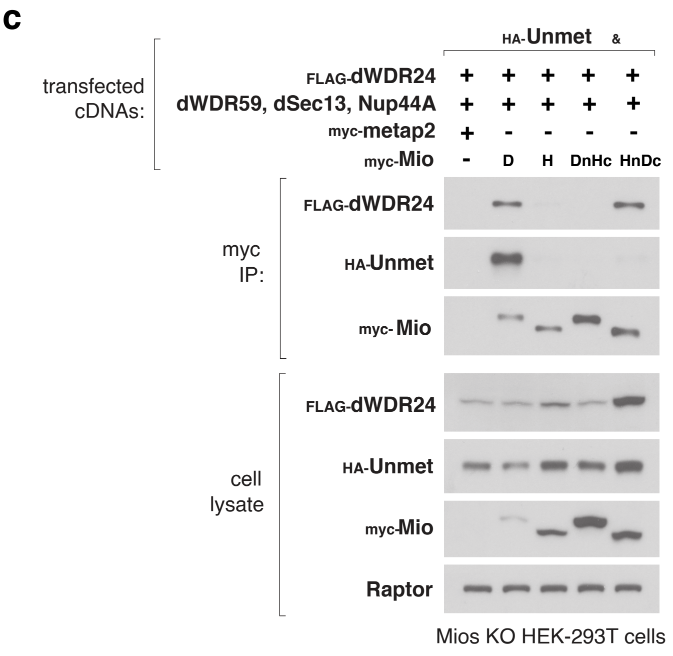

Supplementary Figure 6: The N-terminal WD40 repeat domains of fly GATOR2 mediate species-restricted binding of Unmet.

(**a**) Schematic of the domain structure of Mios homologs in flies and humans. Sequence alignments of Mios from 11 species, with Dipteran species highlighted in yellow. Residues were colored by percent identity and numbered with reference to positions in the human protein sequence. Variable surface-exposed residues conserved in Dipterans and divergent in other species (magenta bars) were mapped onto a structure of human Mios in Fig. 5e.
(**b**) Description of Mio constructs, including fly Mio, human Mios, and Mio chimeras with WDR domain swaps between the human and fly homologs.
(**c**) Substitution of the human N-terminal WDR domain of Mios into the fly Mio protein maintains the integrity of the dGATOR2 complex but abolishes binding to Unmet. HEK-293T cells lacking human Mios were co-transfected with the indicated cDNAs, and anti-myc immunoprecipitates were analyzed by immunoblotting for the indicated proteins and epitope tags. Mio HnDc, shown schematically in (b), contains the human WD40 repeat region. It fails to interact with Unmet but maintains dGATOR2 formation by binding to dWDR24.

a

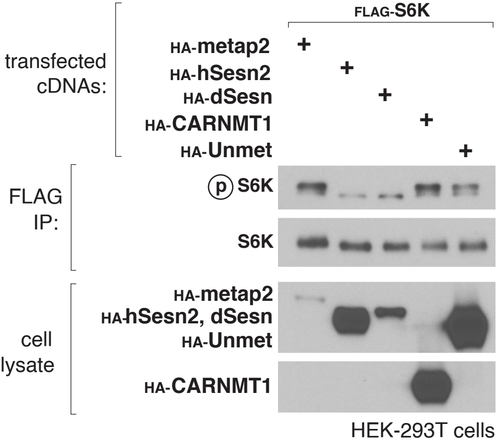
FLAG-S6K
transfected cDNAs:
HA-metap2
HA-hSesn2
HA-dSesn
HA-CARNMT1
HA-Unmet
+
+
+
+
+
FLAG IP:
p S6K
S6K
cell lysate
HA-metap2
HA-hSesn2, dSesn
HA-Unmet
HA-CARNMT1
HEK-293T cells

Supplementary Figure 7: Overexpression of Unmet homologs fails to suppress mTORC1 in human cells.

(**a**) Overexpression of Unmet and its human homolog, CARNMT1, in human cells fails to inhibit mTORC1 signaling. Anti-FLAG immunoprecipitates were analyzed by immunoblotting for the indicated proteins and epitope tags.

Supplementary Table 1: Expression constructs for main figures

**Figure 2:**	
b)	
pGL1-HA-Und	
pGL1-HA-dSesn	
pGL1-HA-Unmet	
c)	
pRK5-FLAG-Iml1	FLAG-dGATOR1
pRK5-myc-dNprl2	
pRK5-myc-dNprl3	
pRK5-FLAG-dWDR59	FLAG-dGATOR2
pRK5-myc-dWDR24	
pRK5-myc-Mio	
pRK5-myc-Nup44A	
pRK5-myc-dSec13	
pRK5-FLAG-Depdc5	FLAG-hGATOR1
pRK5-myc-Nprl2	
pRK5-myc-Nprl3	
pRK5-FLAG-WDR59	FLAG-hGATOR2
pRK5-myc-WDR24	
pRK5-myc-Mios	
pRK5-myc-Sec13	
pRK5-myc-Seh1L	
pRK5-HA-Und	
pRK5-HA-dSesn	
pRK5-HA-Unmet	
d)	
pRK5-FLAG-dWDR59	FLAG-dGATOR2
pRK5-myc-WDR24	
pRK5-myc-Mio	
pRK5-myc-Nup44A	
pRK5-myc-dSec13	
pRK5-HA-Und	
pRK5-HA-dSesn	
pRK5-HA-Unmet	
e)	
pRK5-FLAG-WDR59	FLAG-hGATOR2
pRK5-myc-WDR24	
pRK5-myc-Mios	
pRK5-myc-Sec13	
pRK5-myc-Seh1L	
pRK5-FLAG-dWDR59	FLAG-dGATOR2
pRK5-myc-WDR24	
pRK5-myc-Mio	
pRK5-myc-Nup44A	
pRK5-myc-dSec13	
pRK5-HA-metap2	
pRK5-CASTOR1-HA	

pRK5-HA-dSesn	
pRK5-HA-Unmet	
**Figure 3:**	
d)	
pRK5-FLAG-dWDR59	FLAG-dGATOR2
pRK5-myc-WDR24	
pRK5-myc-Mio	
pRK5-myc-Nup44A	
pRK5-myc-dSec13	
pRK5-HA-Unmet	
pRK5-HA-Unmet G195D	
e)	
pRK5-FLAG-dWDR59	FLAG-dGATOR2
pRK5-myc-WDR24	
pRK5-myc-Mio	
pRK5-myc-Nup44A	
pRK5-myc-dSec13	
pRK5-HA-metap2	
pRK5-HA-Unmet	
pRK5-HA-Unmet E30A	
f)	
pAc5-3xFLAG-Unmet G195D-STABLE2	
pAc5-3xFLAG-Unmet E380A-STABLE2	
**Figure 5:**	
a)	
pRK5-HA-CARNMT1	
pRK5-FLAG-metap2	
pRK5-FLAG-Iml1	FLAG-dGATOR1
pRK5-myc-dNprl2	
pRK5-myc-dNprl3	
pRK5-FLAG-dWDR59	FLAG-dGATOR2
pRK5-myc-dWDR24	
pRK5-myc-Mio	
pRK5-myc-Nup44A	
pRK5-myc-dSec13	
pRK5-FLAG-Depdc5	FLAG-hGATOR1
pRK5-myc-Nprl2	
pRK5-myc-Nprl3	
pRK5-FLAG-WDR59	FLAG-hGATOR2
pRK5-myc-WDR24	
pRK5-myc-Mios	
pRK5-myc-Sec13	
pRK5-myc-Seh1L	
b)	
pRK5-FLAG-*S. pombe* CARNMT1	
pRK5-HA-metap2	
pRK5-HA-dWDR59	HA-dGATOR2
pRK5-myc-dWDR24	
pRK5-myc-Mio	

<table>
<tr><td>pRK5-myc-Nup44A</td><td rowspan="2"></td></tr>
<tr><td>pRK5-myc-dSec13</td></tr>
<tr><td>pRK5-HA-SEA3</td><td rowspan="5">HA-S. pombe SEACAT</td></tr>
<tr><td>pRK5-myc-SEA2</td></tr>
<tr><td>pRK5-myc-SEA4</td></tr>
<tr><td>pRK5-myc-S. pombe Sec13</td></tr>
<tr><td>pRK5-myc-S. pombe Seh1</td></tr>
<tr><td colspan="2">d)</td></tr>
<tr><td colspan="2">See Supplementary Table 2 for constructs used in Supplementary Fig. 5b.</td></tr>
<tr><td colspan="2">Figure 6:</td></tr>
<tr><td colspan="2">a)</td></tr>
<tr><td>pRK5-FLAG-S6K1</td><td></td></tr>
<tr><td>pRK5-HA-dWDR59</td><td rowspan="5">HA-dGATOR2</td></tr>
<tr><td>pRK5-myc-dWDR24</td></tr>
<tr><td>pRK5-myc-Mio</td></tr>
<tr><td>pRK5-myc-Nup44A</td></tr>
<tr><td>pRK5-myc-dSec13</td></tr>
<tr><td>pRK5-HA-metap2</td><td></td></tr>
<tr><td>pRK5-HA-dSesn</td><td></td></tr>
<tr><td>pRK5-HA-Unmet</td><td></td></tr>
<tr><td>pRK5-HA-CARNMT1</td><td></td></tr>
</table>

Supplementary Table 2: Expression constructs for supplementary figures

**Supplementary Figure 2:**	
a)	
pRK5-HA-Und	
pRK5-HA-dSesn	
pRK5-HA-Unmet	
b)	
pGL2-HA-Unmet	
pGL2-HA-dWDR59	
c)	
pRK5-FLAG-dWDR59	FLAG-dGATOR2
pRK5-myc-WDR24	
pRK5-myc-Mio	
pRK5-myc-Nup44A	
pRK5-myc-dSec13	
pRK5-HA-Unmet	
**Supplementary Figure 3:**	
e)	
pGL1-HA-Und	
pGL1-HA-dSesn	
pGL1-HA-dSAMTOR	
**Supplementary Figure 5:**	
a)	
pRK5-HA-Unmet	
pRK5-FLAG-metap2	
pRK5-FLAG-dWDR24	
pRK5-myc-Nup44A	
pRK5-myc-Mio	
pRK5-myc-dWDR59	
pRK5-dSec13	
b)	
pRK5-HA-*D. simulans* WDR24	
pRK5-HA-*D. simulans* Mio	
pRK5-HA-*D. simulans* Nup44A	
pRK5-HA-*D. elegans* WDR24	
pRK5-HA-*D. elegans* Mio	
pRK5-HA-*D. elegans* Nup44A	
pRK5-HA-*D. busckii* WDR24	
pRK5-HA-*D. busckii* Mio	
pRK5-HA-*D. busckii* Nup44A	
pRK5-HA-*L. cuprina* WDR24	
pRK5-HA-*L. cuprina* Mio	
pRK5-HA-*L. cuprina* Nup44A	
pRK5-HA-*A. aegypti* WDR24	
pRK5-HA-*A. aegypti* Mio	
pRK5-HA-*A. aegypti* Nup44A	
pRK5-HA-*A. mellifera* WDR24	
pRK5-HA-*A. mellifera* Mio	

pRK5-HA-*A. mellifera* Nup44A	
pRK5-HA-*D. pulex* WDR24	
pRK5-HA-*D. pulex* Mio	
pRK5-HA-*D. pulex* Nup44A	
pRK5-HA-*D. melanogaster* WDR24	
pRK5-HA-*D. melanogaster* Mio	
pRK5-HA-*D. melanogaster* Nup44A	
pRK5-HA-*B. floridae* WDR24	
pRK5-HA-*B. floridae* Mio	
pRK5-HA-*B. floridae* Nup44A	
pRK5-HA-*C. milii* WDR24	
pRK5-HA-*C. milii* Mio	
pRK5-HA-*C. milii* Nup44A	
pRK5-FLAG-metap2	
pRK5-FLAG-*D. simulans* CARNMT1	
pRK5-FLAG-*D. elegans* CARNMT1	
pRK5-FLAG-*D. busckii* CARNMT1	
pRK5-FLAG-*L. cuprina* CARNMT1	
pRK5-FLAG-*A. aegypti* CARNMT1	
pRK5-FLAG-*A. mellifera* CARNMT1	
pRK5-FLAG-*D. pulex* CARNMT1	
pRK5-FLAG-*D. melanogaster* CARNMT1 (Unmet)	
pRK5-FLAG-*B. floridae* CARNMT1	
pRK5-FLAG-*C. milii* CARNMT1	
**Supplementary Figure 6:**	
c)	
pRK5-HA-Unmet	
pRK5-FLAG-dWDR24	
pRK5-dWDR59	
pRK5-dSec13	
pRK5-myc-metap2	
pRK5-myc-Mio (D)	
pRK5-myc-Mios (H)	
pRK5-myc-Mio DnHc	
pRK5-myc-Mio HnDc	
**Supplementary Figure 7:**	
a)	
pRK5-FLAG-S6K1	
pRK5-HA-metap2	
pRK5-HA-hSesn2	
pRK5-HA-dSesn	
pRK5-HA-CARNMT1	
pRK5-HA-Unmet	
