# Supplementary material for: An evolutionary mechanism to assimilate new nutrient sensors into the mTORC1 pathway
Source: Nat Commun. 2024 Mar 21;15:2517. doi: 10.1038/s41467-024-46680-3 (PMC10957897; doi:10.1038/s41467-024-46680-3)
Supplement: Supplementary file 3 — Reporting Summary [file 41467_2024_46680_MOESM3_ESM.pdf]

nature portfolio

Corresponding author(s): Grace Y. Liu
Norbert Perrimon
David M. Sabatini

Last updated by author(s): Dec 28, 2023

Reporting Summary

Nature Portfolio wishes to improve the reproducibility of the work that we publish. This form provides structure for consistency and transparency in reporting. For further information on Nature Portfolio policies, see our Editorial Policies and the Editorial Policy Checklist.

Statistics

For all statistical analyses, confirm that the following items are present in the figure legend, table legend, main text, or Methods section.

n/a	Confirmed	
☐	☒	The exact sample size (*n*) for each experimental group/condition, given as a discrete number and unit of measurement
☐	☒	A statement on whether measurements were taken from distinct samples or whether the same sample was measured repeatedly
☐	☒	The statistical test(s) used AND whether they are one- or two-sided<br>*Only common tests should be described solely by name; describe more complex techniques in the Methods section.*
☒	☐	A description of all covariates tested
☐	☒	A description of any assumptions or corrections, such as tests of normality and adjustment for multiple comparisons
☐	☒	A full description of the statistical parameters including central tendency (e.g. means) or other basic estimates (e.g. regression coefficient) AND variation (e.g. standard deviation) or associated estimates of uncertainty (e.g. confidence intervals)
☐	☒	For null hypothesis testing, the test statistic (e.g. *F*, *t*, *r*) with confidence intervals, effect sizes, degrees of freedom and *P* value noted<br>*Give P values as exact values whenever suitable.*
☒	☐	For Bayesian analysis, information on the choice of priors and Markov chain Monte Carlo settings
☒	☐	For hierarchical and complex designs, identification of the appropriate level for tests and full reporting of outcomes
☒	☐	Estimates of effect sizes (e.g. Cohen's *d*, Pearson's *r*), indicating how they were calculated

Our web collection on statistics for biologists contains articles on many of the points above.

Software and code

Policy information about availability of computer code

Data collection	Zeiss ZEN Black 2012, QuantStudio6 RT-PCR
Data analysis	Clustal Omega 1.2.4, GraphPad Prism 8.2.1, Fiji with ImageJ 2.1.0/1.53c, Microsoft Excel 2019 v.1808, Dendroscope 3.8.4, RAxML 1.0.0

For manuscripts utilizing custom algorithms or software that are central to the research but not yet described in published literature, software must be made available to editors and reviewers. We strongly encourage code deposition in a community repository (e.g. GitHub). See the Nature Portfolio guidelines for submitting code & software for further information.

Data

Policy information about availability of data

All manuscripts must include a data availability statement. This statement should provide the following information, where applicable:

- Accession codes, unique identifiers, or web links for publicly available datasets
- A description of any restrictions on data availability
- For clinical datasets or third party data, please ensure that the statement adheres to our policy

The data generated in this study are provided in the Source Data file and are available from the corresponding authors upon request. Homologs of mTOR (DROME01015), Unmet (DROME30051), WDR24 (DROME19416), Mios (DROME01365), and Seh1L (DROME05734) were drawn from the OMA Orthology Database. Plasmids generated in this study are available on Addgene.

Research involving human participants, their data, or biological material

Policy information about studies with human participants or human data. See also policy information about sex, gender (identity/presentation), and sexual orientation and race, ethnicity and racism.

Reporting on sex and gender	N/A
Reporting on race, ethnicity, or other socially relevant groupings	N/A
Population characteristics	N/A
Recruitment	N/A
Ethics oversight	N/A

Note that full information on the approval of the study protocol must also be provided in the manuscript.

Field-specific reporting

Please select the one below that is the best fit for your research. If you are not sure, read the appropriate sections before making your selection.

☒ Life sciences ☐ Behavioural & social sciences ☐ Ecological, evolutionary & environmental sciences

For a reference copy of the document with all sections, see nature.com/documents/nr-reporting-summary-flat.pdf

Life sciences study design

All studies must disclose on these points even when the disclosure is negative.

Sample size	No statistical method was used to pre-determine the sample size. Based on previous experience and standards in the field, we used a minimal sample size of n = 3 for quantitative experiments.
Data exclusions	No data were excluded from the analyses.
Replication	All experimental findings were repeated at least two times.
Randomization	Flies were randomly allocated into experimental groups. For all other experiments, samples were processed in parallel and measured independently, rendering group allocation and randomization unnecessary.
Blinding	Investigators were blinded to group allocation of flies during analysis and scoring of stained ovaries.

Reporting for specific materials, systems and methods

We require information from authors about some types of materials, experimental systems and methods used in many studies. Here, indicate whether each material, system or method listed is relevant to your study. If you are not sure if a list item applies to your research, read the appropriate section before selecting a response.

Materials & experimental systems

n/a	Involved in the study	
☐	☒	Antibodies
☐	☒	Eukaryotic cell lines
☒	☐	Palaeontology and archaeology
☐	☒	Animals and other organisms
☒	☐	Clinical data
☒	☐	Dual use research of concern
☒	☐	Plants

Methods

n/a	Involved in the study	
☒	☐	ChIP-seq
☒	☐	Flow cytometry
☒	☐	MRI-based neuroimaging

Antibodies

Antibodies used	Mouse anti-FLAG (M2), Millipore Sigma (F1804); Rabbit anti-Raptor, EMD Millipore (09-217); Rabbit anti-Depdc5, Abcam (ab185565); Rabbit anti-β-actin, CST (4967); Rabbit anti-phospho-T398 dS6K, CST (9209); Rabbit anti-cleaved Drosophila Dcp-1 Asp216, CST (9578); Rabbit anti-Mios, CST (13557); Rabbit anti-FLAG epitope, CST (14793); Rabbit anti-HA epitope, CST (3724); Rabbit anti-myc

	epitope, CST (2278); Mouse anti-hu-li tai shao, DSHB (1B1); Donkey Alexa 488-conjugated anti-rabbit secondary antibody, Thermo Fisher Scientific (A-21206); Goat Alexa 555-conjugated anti-mouse secondary antibody, Thermo Fisher Scientific (A28180); HRP-conjugated anti-mouse IgG secondary antibody, CST (7076); HRP-conjugated anti-rabbit IgG secondary antibody, CST (7074); Rabbit anti-dS6K, gift from Mary Stewart (North Dakota State University). For Western blots, all primary antibodies were used at a dilution of 1:1000, with the exception of the anti-dS6K antibody, which was used at 1:10,000. Secondary HRP-conjugated antibodies were used at a dilution of 1:3000 for Western blots.
Validation	All antibodies were validated by the manufacturer according to provided materials. All antibodies have been previously published.

Eukaryotic cell lines

Policy information about cell lines and Sex and Gender in Research

Cell line source(s)	HEK-293T cells were obtained from ATCC. S2R+ cells were obtained from the Perrimon lab.
Authentication	None
Mycoplasma contamination	No mycoplasma contamination was detected by PCR.
Commonly misidentified lines (See ICLAC register)	N/A

Animals and other research organisms

Policy information about studies involving animals; ARRIVE guidelines recommended for reporting animal research, and Sex and Gender in Research

Laboratory animals	D. melanogaster strains (y,sc,v; nos-Cas9) were provided by the Bloomington Drosophila Stock Center. Experiments were conducted on synchronized L3 larvae or 5-day-old female adults.
Wild animals	The study did not involve wild animals.
Reporting on sex	Findings relating to ovarian degeneration apply only to adult female flies, as these organs are absent in male flies and larvae. Sex of eclosed flies was assigned based on morphological differences between male and female flies.
Field-collected samples	The study did not involve samples collected from the field.
Ethics oversight	N/A

Note that full information on the approval of the study protocol must also be provided in the manuscript.

Plants

Seed stocks	*Report on the source of all seed stocks or other plant material used. If applicable, state the seed stock centre and catalogue number. If plant specimens were collected from the field, describe the collection location, date and sampling procedures.*
Novel plant genotypes	*Describe the methods by which all novel plant genotypes were produced. This includes those generated by transgenic approaches, gene editing, chemical/radiation-based mutagenesis and hybridization. For transgenic lines, describe the transformation method, the number of independent lines analyzed and the generation upon which experiments were performed. For gene-edited lines, describe the editor used, the endogenous sequence targeted for editing, the targeting guide RNA sequence (if applicable) and how the editor was applied.*
Authentication	*Describe any authentication procedures for each seed stock used or novel genotype generated. Describe any experiments used to assess the effect of a mutation and, where applicable, how potential secondary effects (e.g. second site T-DNA insertions, mosiacism, off-target gene editing) were examined.*
